# An Investigation of Rotary Drone HERM Line Spectrum under Manoeuvering Conditions

**DOI:** 10.3390/s20205940

**Published:** 2020-10-21

**Authors:** Peter Klaer, Andi Huang, Pascale Sévigny, Sreeraman Rajan, Shashank Pant, Prakash Patnaik, Bhashyam Balaji

**Affiliations:** 1 Defence Research and Development Canada, 3701 Carling Avenue, Ottawa, ON K2K 2Y7, Canada; peterklaer@gmx.de (P.K.); AndiHuang@cmail.carleton.ca (A.H.); pascale.sevigny@drdc-rddc.gc.ca (P.S.); 2 Department of Systems and Computer Engineering, Carleton University, 1125 Colonel By Drive, Ottawa, ON K1S 5B6, Canada; sreeramanr@sce.carleton.ca; 3 Aerospace Research Centre, National Research Council Canada, 1200 Montreal Rd., Ottawa, ON K1A 0R6, Canada; shashank.pant@nrc-cnrc.gc.ca (S.P.); prakash.patnaik@nrc-cnrc.gc.ca (P.P.)

**Keywords:** micro-Doppler, UAV, drone, radar, HERM lines, spool lines, chopping lines, log harmonic summation, multi-frequency analysis

## Abstract

Detecting and identifying drones is of great interest due to the proliferation of highly manoeuverable drones with on-board sensors of increasing sensing capabilities. In this paper, we investigate the use of radars for tackling this problem. In particular, we focus on the problem of detecting rotary drones and distinguishing between single-propeller and multi-propeller drones using a micro-Doppler analysis. Two different radars were used, an ultra wideband (UWB) continuous wave (CW) C-band radar and an automotive frequency modulated continuous wave (FMCW) W-band radar, to collect micro-Doppler signatures of the drones. By taking a closer look at HElicopter Rotor Modulation (HERM) lines, the spool and chopping lines are identified for the first time in the context of drones to determine the number of propeller blades. Furthermore, a new multi-frequency analysis method using HERM lines is developed, which allows the detection of propeller rotation rates (spool and chopping frequencies) of single and multi-propeller drones. Therefore, the presented method is a promising technique to aid in the classification of drones.

## 1. Introduction

The detection of drones using radars at long ranges is a difficult problem due to their small size and small radar cross sections. In addition, the misidentification of drones as birds is a frequent occurrence because birds can have similar radar cross-sections as certain types of drones. Micro-Doppler analysis provides a way to distinguish between different types of flying objects by examining the frequency modulation of the Doppler frequency caused by micro motions of the objects, such as the movement of wings or rotating propellers [1,2,3,4].

The classification of drones via radar signatures using micro-Doppler information has been investigated using a variety of techniques [5,6]. Some approaches include using features derived from spectrograms [7], the application of deep learning techniques to range-Doppler maps [8], and cadence–velocity diagrams [9], as well as polarimetry [10].

Micro-Doppler radar signatures are studied using time-frequency analysis, and most commonly using the Short Time Fourier Transform (STFT) [2,3,5,6,11,12,13,14,15]. Depending on the STFT window length, two different types of spectrograms are obtained. If the window length is shorter than the rotation period of the propeller and the pulse repetition frequency (PRF) of the radar is sufficiently high, then it is possible to capture the periodic behavior of the individual rotating blades, referred to as blade flashes [16]. The spectrogram can provide information on the number of rotating rotor blades and their relative direction of motion.

Applying a long window length (integration over several cycles of the rotor blades rotation) for STFT shrinks the time resolution, but increases the frequency resolution, which reveals HElicopter Rotor Modulation (HERM) lines in the spectrogram plot [2,3,4]. HERM lines are spectral lines that are periodic in Doppler frequency rather than time. They are separated by a quantity that is a function of the rotation rate and the number of rotor blades, among other factors.

From the Nyquist–Shannon sampling considerations, the Pulse Repetition Frequency (PRF) of a radar needs to be at least four times the maximum Doppler shift induced by the rotating propellers in order to resolve micro-Doppler signatures with blade flashes [1]. This requirement can be difficult to meet for some radars, such as an airport surveillance radar. In the situation where the radar PRF is insufficient, a long window STFT can still be used to extract micro-Doppler signatures from HERM lines. For HERM lines, the radar PRF only has to be at least twice the propeller rotation rate to fulfill the Nyquist–Shannon sampling theorem.

In this paper, we analyze the HERM lines produced by rotary drones. The novelty of our approach is that the HERM lines produced by rotary drones are discussed in terms of two classes of spectral lines, namely the chopping lines and spool lines. The spool lines are directly related to the propeller rotation rate, while the chopping lines result from the multiplicity of blades. Spool and chopping lines have been investigated only in the context of Jet Engine Modulation (JEM) lines of airplanes [17], but not in the context of drones. The analysis of the chopping and spool lines in the context of drones offers the possibility of determining the number of blades and the size of the propeller, which can help in the identification of types of drones.

Since HERM lines are periodic in frequency, the detection of HERM lines essentially becomes a pitch detection problem where pitch is the fundamental frequency of the HERM lines. In this paper, the recently proposed log harmonic summation algorithm from [18] is extended to a novel multi-frequency detector. The multi-frequency detector offers the possibility of estimating the chopping and spool frequencies of a single-propeller drone, or even the chopping and spool frequencies of an individual propeller of a multi-propeller drone. This allows the possibility to classify the drone as a one-propeller or multi-propeller drone, and also to distinguish a drone from a bird.

Results of the multi-frequency analysis are presented for different types of drones in manoeuvering scenarios emulated in a laboratory setting and measured with low power software-defined radars in the C and W-band. This paper forms the basis for future analysis of multi-frequency detector, distinguishing drones from birds, and classification of drones.

The paper is arranged in the following manner. A discussion of three types of drones used in our experiments, the experimental set up, and the required fundamentals of micro-Doppler processing, and the log harmonic summation to understand this paper are presented in Section 2. The experimental results obtained using two types of radars used in this study are analyzed in Section 3 along with the proposed multi-frequency detection algorithm, which is followed by the conclusion in Section 4.

## 2. Materials and Methods

### 2.1. Experimental Setup

Two different radars were used to collect micro-Doppler signatures of drones, namely the C-band Xethru X4 UWB pulsed CW radar and the W-band TI AWR1642 automotive FMCW radar. The Xethru X4 radar operates at 7.29 GHz and has a maximum Pulse Repetition Frequency (PRF) of only 1.5 kHz. The TI AWR1642 radar operates at 77 GHz, and, as such, is reserved for automotive radars. In this paper, it is used as an illustrative example of the potential utility of higher operating frequencies and higher PRFs in this problem. This radar has a higher bandwidth and with a radar PRF of 26.3 kHz. The C-band radar has a lower range than the TI radar. The range resolution of C-band is higher than that of the TI radar. Table 1 lists the specifications of both radars, where TX denotes the transmitted pulse and RX the received pulse of the radar.

### 2.2. Drone Properties and Manoeuvering Characteristics

**Figure 1:**
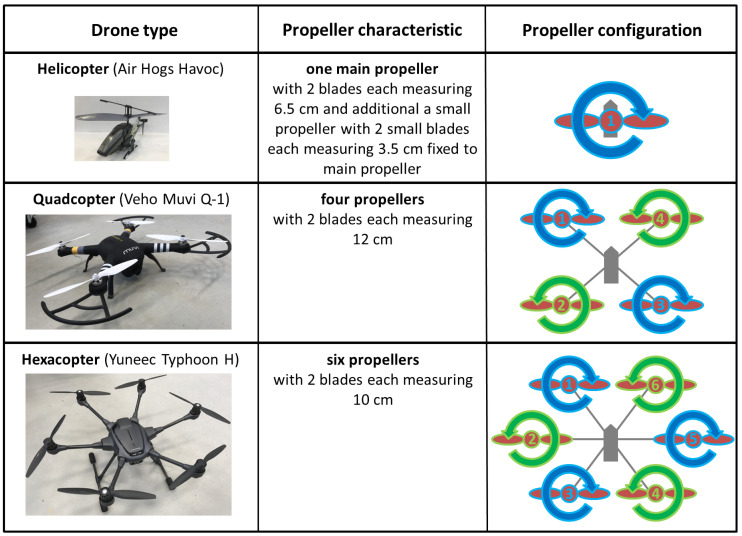
Drone propeller characteristics and configurations.

Figure 1 illustrates the drones used along with their propeller characteristics and configurations.

The helicopter drone has a one-propeller configuration, which requires an additional small tail propeller to compensate the main propeller torque. Since this tail propeller is very small, the contribution to the micro-Doppler spectrum is negligible.

For the quadcopter, two propellers (1 and 3) are rotating clockwise (cw) and two propellers (2 and 4) are rotating counterclockwise (ccw) in a four-propeller configuration. In a hovering situation (assuming windless conditions), all propellers have the same rotation rate and the lift force on all propellers equals the gravitational force of the drone. Increasing or decreasing the propeller rotation rate of all propellers lifts or lowers the drone. Accelerating one cw and one ccw propeller and decelerating the other two cw and ccw propellers moves the drone to the direction of the lowest lift. For example, by accelerating propellers 1 and 2 and decelerating propellers 3 and 4, the drone moves to the right. Accelerating the two cw propellers (1 and 3) and decelerating the ccw propellers (2 and 4) causes a torque, and the drone rotates cw.

The hexacopter has a six-propeller configuration. Similar to quadcopters, all propellers have the same rotation rate in a hovering situation. To lift or to lower the drone, all propellers accelerate or decelerate, respectively. With propellers 3 and 4 accelerating and propellers 1 and 6 decelerating, the drone moves forward due to the higher lift at the back part of the drone. Accelerating propeller 2 and decelerating propeller 5 while keeping the other propellers at the same rotation rate moves the drone to the right. A cw and a ccw drone rotation torque can be achieved by the difference of the rotation rate of all cw and all ccw propellers.

Of course, in a real hovering or manoeuvering scenario, e.g., in windy conditions, all propellers might have individual rotation rates. However, the above discussion suggests an approach to investigate some aspects of manoeuvering drones in laboratory conditions.

### 2.3. Basic Concept of Micro-Doppler

The micro-Doppler effect occurs when there is mechanical vibration or motion of an object along the Line of Sight (LoS) of the radar, which induces additional frequency modulation on the returned signal beyond the main body Doppler frequency. This generates sidebands about the Doppler frequency shift of the main body [11].

**Figure 2:**
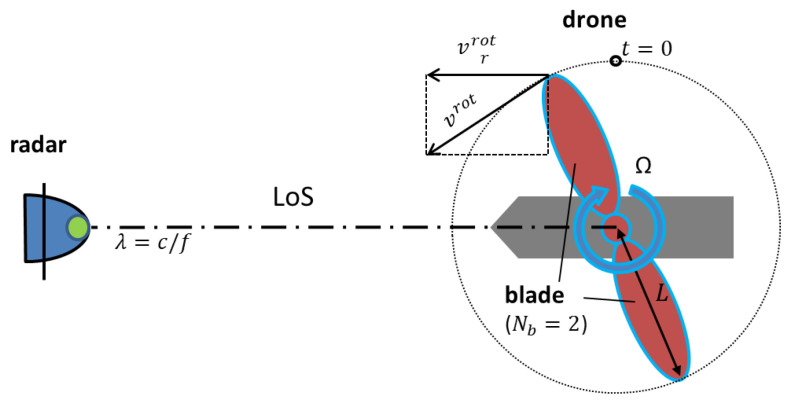
Schematic of micro-Doppler measurement in a simple model.

For a single propeller, shown in Figure 2, with a rotation axis perpendicular to the LoS, the micro-Doppler shift $f_{D}^{rot}$ is determined by the radar wavelength λ and the relative or radial blade tip speed $v_{r}^{rot}$ as

(1) $$f_{D}^{rot}=-\frac{2}{\lambda }v_{r}^{rot}\;.$$

The radial blade tip velocity $v_{r}^{rot}=v^{rot}\cos\left(2\pi \Omega t\right)$ is the velocity of the blade tip along the LoS of the radar with respect to the tangential velocity $v^{rot}$, where Ω is the rotation rate and *t* is the time. The highest $v_{r}^{rot}$ is reached when the blades are perpendicular to LoS. A positive micro-Doppler frequency shift is caused by an approaching blade and a negative micro-Doppler frequency shift is caused by a receding blade.

The blade tip velocity can be expressed as

(2) $$v^{rot}=2\pi L\Omega \;,$$

where *L* is the blade length.

As an example, for Xethru C-band radar, maximum unambiguous velocity of a propeller tip of 15.32 m/s can be obtained.

A popular time-frequency method to extract micro-Doppler signatures from the radar data are the Short Time Fourier Transform (STFT). STFT of the radar returns at given range of the drone is given as

(3) $$X\left[n,\omega \right]=\sum_{m=-\infty }^{\infty }x\left[m\right]w\left[m-n\right]e^{-j\omega m}$$

where *w* is a tapered window that controls the time resolution, and *n* gives the time-shift of the window. If the PRF of the radar is sufficient, the short window STFT can be used to show the individual blade flashes in a time-frequency spectrogram. In literature, the term time-velocity diagram is sometimes used instead of the time-frequency spectrogram because of the relation provided by Equation (1).

A long window STFT can be used as an alternative way to visualize micro-Doppler. By applying a longer window, several cycles of the rotor blades are included in the coherent processing interval (CPI), and the spectrogram is dominated by the rotation frequency of the blades causing modulation peaks and the instantaneous relative velocity $v_{r}^{rot}$ of the blades is no longer observable [2]. The long window STFT results in a so-called HElicopter Rotor Modulation (HERM) lines spectrogram.

### 2.4. Log Harmonic Summation

The log harmonic summation method was used to extract the fundamental frequency from the HERM lines in Ref. [18]. It was shown that the method is appropriate even when the signal-to-noise ratio is very low. The log harmonic summation algorithm is a technique borrowed from pitch estimation that uses a pattern matching technique to sum up the *n* harmonics of every frequency in a signal [19]. This algorithm essentially measures the strength of harmonics for every frequency. The frequency with the strongest magnitude is selected as the fundamental frequency. This is applicable to HERM lines since they are spectral lines that repeat harmonically at some frequency.

**Figure 3:**
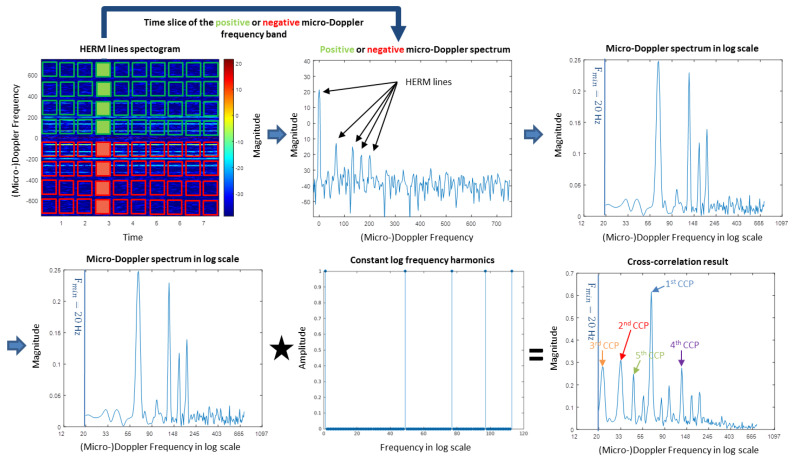
Steps of the log harmonic summation algorithm applied on a HERM lines spectrogram.

The steps for the log harmonic summation algorithm outlined in Figure 3 are as follows:

1. Generate a HERM lines spectrogram (long window STFT) with the body line shifted to 0 Hz and with an appropriate STFT window size. For the C-band radar, a window size of 512 samples and for the W-band radar, a window size of 4096 samples with an overlap of 80% are used.
2. Choose the positive or negative micro-Doppler frequency band for further analysis. In the following, each HERM line’s time slice (micro-Doppler spectrum) has to be processed separately. The window size of the positive or the negative micro-Doppler spectrum is half of the number of window size used for the long window STFT. Hence, for the C-band and the W-band, the micro-Doppler frequency lengths are 256 samples and 2048 samples, respectively. The size of a time bin is given by the radar PRF and the window size chosen for the STFT; thus, for a C-band radar with a PRF of 1500 Hz, the time window size is 0.34 s and for W-band radar with a PRF of 26,316 Hz, the time window size is 0.156 s.
3. Interpolate the micro-Doppler spectrum to get a constant log frequency spacing for the cross-correlation step. In this paper, the minimum frequency is set to 20 Hz and 48 bins per octave are used. The minimum frequency is chosen as 20 Hz so that contributions of the main body line and low frequency micro-Doppler components can be ignored. Forty-eight bins per octave are used to generate 202 log frequency points for the C-band radar, requiring an interpolation from 256 to 202 points. For the W-band radar, 48 bins per octave generates 450 log frequency points leading to an interpolation from 2048 to 450 points. For the W-band radar, some resolution loss occurs at higher frequencies, but this is acceptable since we are expecting a fundamental frequency under 300 Hz.
4. Apply a cross-correlation to the micro-Doppler spectrum in log scale and the constant log frequency harmonics at every frequency. For the cross-correlation results of the C-band radar, three harmonics and, of the W-band radar, five harmonics at every frequency are used. The strongest Cross-Correlation Peak (1st CCP) determines the fundamental frequency.

## 3. Experimental Results

In this section, radar measurements with the C-band and W-band radars with time-varying rotation frequencies are performed in a laboratory condition are discussed, and the results are analyzed. The features of HERM lines spectrogram are considered drawing the motivation from the JEM spectrogram [17].

The radars were positioned between 50 cm and 1 m away in front of the drones. For these measurements, the drones were fixed to a stand. This means that any manoeuvering drone scenario shown was done without a real physical movement of the drone body; instead, the propeller rotation rates were varied to emulate some aspects of drone manoeuvrability. The rotor rotation speeds were adjusted to simulate manoeuvering. Therefore, the Doppler frequency of the body line (at 0 Hz) in the HERM lines spectrograms does not change in this emulated manoeuvering scenario. In a real drone flying scenario, the Doppler frequency of the body line shift with respect to the moving direction and the HERM lines would shift accordingly. In addition, there are complications due to range migration, which are not considered here.

### 3.1. Spool and Chopping Lines

**Figure 4:**
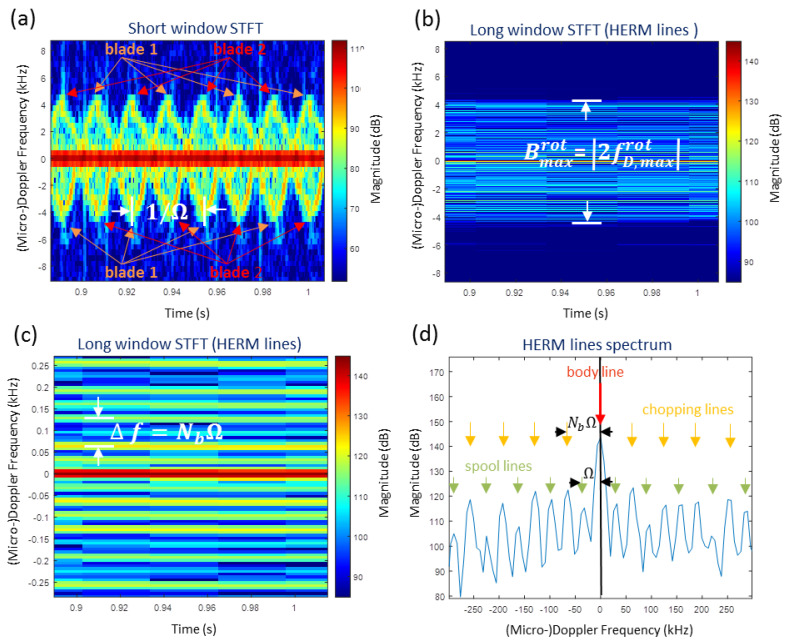
Example of a measured micro-Doppler spectrogram of the coaxial helicopter by W-band radar using (**a**) short window STFT and (**b**) long window STFT; (**c**) zoomed into the long window STFT showing individual HERM lines; (**d**) HERM lines spectrum at 1 s.

Figure 4 compares a short window STFT spectrogram with a long window STFT spectrogram of the coaxial helicopter. A sinusiodal spectrogram for the two blade propeller is observed for the short window STFT showing individual propeller blade flashes. The long window STFT leads to HERM lines.

In Figure 4d, the HERM lines spectrum from Figure 4c centered at 1 s is shown. The positions of the body line, the spool lines, and the chopping lines are indicated by small arrows. The chopping and spool lines appear due to the net effect of all the contributions of the rotor blades reflecting the signal back in the direction of the radar [17].

The body line is caused by the radar cross-section of the main body, whereas the spool and the chopping frequency are caused by the cross-section of the rotor blades. Typically, the magnitude of the body line is found to be approximately 20 dB higher than the chopping lines and the spool lines are weaker than the chopping lines. The frequency difference of the first harmonic chopping line and the body line or the frequency difference of two adjacent lines indicate the chopping frequency $f_{C}=N_{b}\Omega$, which is the rotation frequency of the propeller, where $N_{b}$ is the number of blades of a propeller. At higher harmonics, the magnitude of the HERM lines decreases.

The spool lines (number of spool lines $=N_{b}-1$) are in between the body line and the first chopping line and also in between two chopping lines. The first spool line in relation to the body line determines the spool frequency $f_{S}=\Omega$ of the propeller, which is the frequency for a $360^{\circ }$ propeller rotation. The spool lines appear because the shape of the blades (screw design) present a different aspect angle to the radar leading to variation of the radar cross-section amplitude measured at the radar over a full cycle [17].

The estimates of spool and chopping frequencies can be used to estimate the number of blades:

(4) $$N_{b}=\frac{f_{C}}{f_{S}}\;.$$

The chopping and spool frequencies can be used to distinguish different flying objects. For example, the chopping frequency of a drone is higher than a flapping frequency of a bird wing, which is typically smaller than 20 Hz [20]. Hence, a measured frequency higher than 20 Hz classifies the flying object as a drone. Another bird and drone distinguishing feature might be that the propeller of a drone generates two interdependent frequencies, the spool and chopping frequency, but a bird will likely only reveal one flapping frequency or two independent flapping frequencies. Furthermore, measuring one or different spool and chopping frequencies allows for determining the number of propellers and the number of blades for each propeller which are characteristic features for drones.

**Figure 5:**
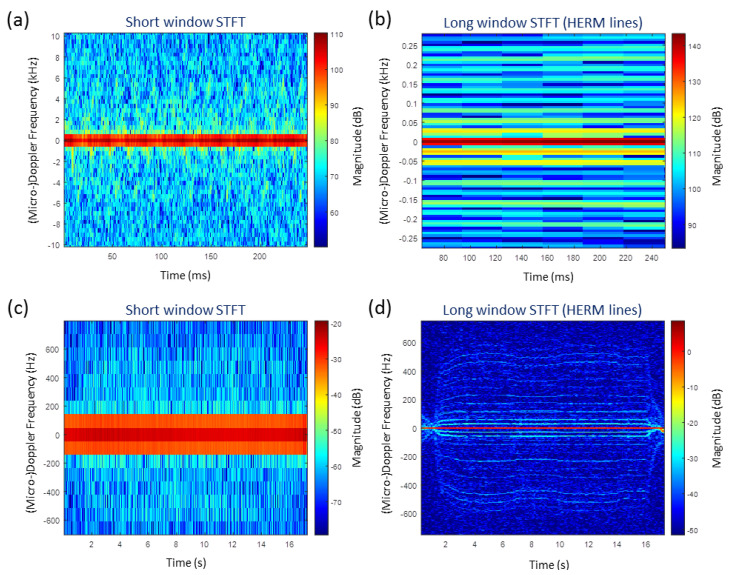
Spectrogram with a low signal to noise ratio of the coaxial helicopter measured by W-band radar using (**a**) short window STFT and (**b**) long window STFT. Spectrogram of the coaxial helicopter measured by C-band radar with insufficient PRF to capture all individual blade flashes using (**c**) short window STFT and (**d**) long window STFT.

The long window STFT (HERM lines) can be used for micro-Doppler drone detection and recognition even if the radar PRF is insufficient or when the signal-to-noise ratio is too low to show all individual blade flashes in a short window STFT. This is illustrated in Figure 5 through two examples. The first one shows the spectrogram measured with a low signal-to-noise ratio by a W-band radar, where the blade flashes are barely visible in the short window STFT while the HERM lines are still visible with the long window STFT. The second example shows the spectrogram measured with a C-band radar where the short window STFT does not show any micro-Doppler feature because the PRF was insufficient relative to the blade tip Doppler frequency, while the long window STFT still produces HERM lines which can be used for analysis. For example, the minimum PRF for a W-band radar (77 GHz) should be approximately 300 kHz for short window STFT and, for long window STFT, a PRF merely of 700 Hz is sufficient. Therefore, even with a low radar PRF, it is possible to extract HERM lines and determine few micro-Doppler features, such as the propeller rotation rate.

### 3.2. Multi-Frequency Detector

The multi-frequency detector is an extension of the log harmonic summation method described before. Instead of determining just one fundamental frequency, it allows for determining multiple frequencies. Up to five frequencies are possible for the multi-frequency detector presented here. This is realized by evaluating not only the strongest cross-correlation peak (1st CCP), but the first five strongest cross-correlation peaks (1st CCP, 2nd CCP, 3rd CCP, 4th CCP and 5th CCP), see Figure 3.

A simple multi-frequency detector algorithm can be devised to determine multiple CCPs from the cross-correlation result of the log harmonic method as follows:

1. Set a minimum threshold for the CCP recognition above the noise level to detect a real CCP and not noise.
2. Find the strongest CCPs of the cross-correlation result of the log harmonic summation. In this paper, only the first five strongest CCPs are used.
3. To establish a harmonic reduction, compare all detected frequencies of all CCPs. If the frequency of the (i + 1)-th CCP is just a higher harmonic of the i-th CCP ignore the result, where i-th CCP indicates here the 1st, 2nd, 3rd, 4th, and 5th CCP. (The current algorithm returns zero for the i-th CCP, if the micro-Doppler frequency time slice does not contain an i-th frequency or when the (i − 1)-th CCP is just a lower harmonic of the i-th CCP.)

Because a HERM lines spectrogram usually contains chopping and spool lines, typically the CCP with higher magnitude is related to the chopping frequency and the CCP with lower magnitude to the spool frequency. For example for a one propeller drone, the 1st CCP properly represents the chopping frequency and the 2nd CCP the spool frequency. This is due to the fact that the magnitude of the spool lines is normally smaller than the magnitude of the chopping lines (see Figure 4d), and this leads to a smaller CCP magnitude.

This algorithm provides an automated method to estimate multiple chopping and spool frequencies from HERM lines’ spectrograms. The estimated values of chopping and the spool frequencies can help distinguish different targets and even distinguish between different types of drones.

However, note that, when a multi-propeller drone is hovering, all propellers might be rotating at the same rotation rate. In that case, the HERM lines will have constant frequency spacing, so that it cannot be distinguished from a one-propeller drone. In addition, for a drone with multiple propellers rotating at different rotation rates, the HERM lines split into multiple paths (non-harmonic HERM lines). When this happens, the simple log harmonic summation algorithm still reports just one fundamental frequency, whereas the multi-frequency detector will report the chopping frequencies of the different propellers.

### 3.3. Multi-Frequency Analysis Using C-Band Radar

In principle, a one-propeller helicopter should produce the simplest HERM lines spectrogram with only one chopping and spool frequency (assuming the tail propeller of the helicopter can be neglected). As the propeller of the drone speeds up (slows down), the chopping and spool frequencies of the HERM lines increases (decreases). There should be no more than one chopping and one spool frequency even if the drone is flying forward, backward or to any other direction.

**Figure 6:**
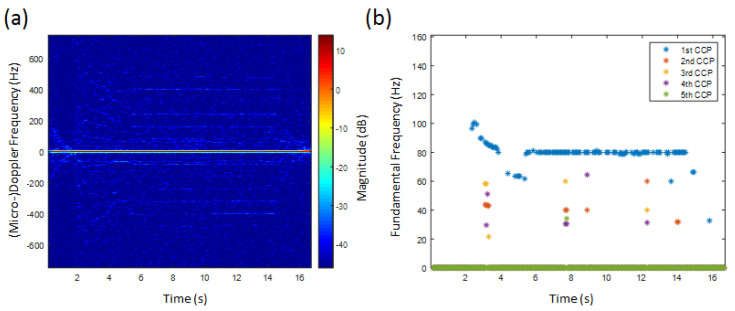
(**a**) HERM lines spectrogram for one-propeller helicopter measured by C-band radar; (**b**) corresponding multi-frequency detector result.

In Figure 6a, the HERM lines spectrogram of a one propeller drone is shown. At about 2 s, the propeller rotation frequency was increased and decreased again to a certain level and manoeuvered to different directions. At 14.5 s, the drone was switched off. Because of the small dimensions of the drone and the radar specifications, the HERM lines appear very weak, and the SNR is very low.

Applying the multi-frequency detector to the HERM lines spectrogram, it is possible to determine the chopping frequency but not the spool frequency of the propeller, see Figure 6b. One possible explanation is that the SNR was too low to expose spool lines. Another explanation could be that the 4 cm radar wavelength is probably not suitable for resolving small 6.5 cm blades due to near field effects. The measured frequency captured by the 1st CCP is the chopping frequency of the drone. Looking at the 1st CCP results, the fundamental frequency was reached at 2.5 s and became nearly constant between 6 s and 14.5 s, even when the manoeuvering direction was changed. The few 2nd to 5th CCP results seem to be false alarms. Because of the high chopping frequency of approximately 80 Hz, which is much higher than the flapping frequency of a bird wing, the possibility of it being a a bird can be excluded. Measuring just one chopping frequency classifies the drone as a one-propeller drone.

**Figure 7:**
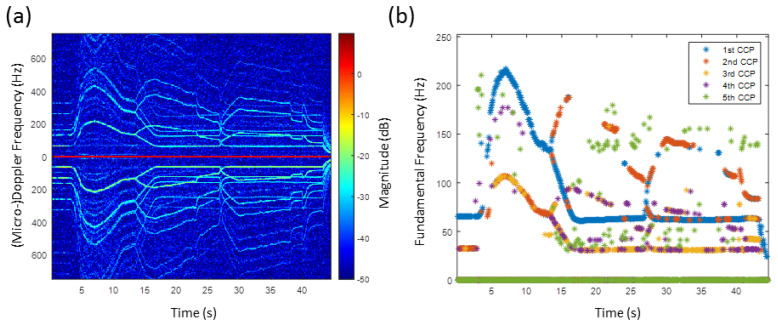
(**a**) HERM lines spectrogram for quadcopter measured by C-band radar; (**b**) corresponding multi-frequency detector result.

Figure 7a shows the HERM lines’ spectrogram for a quadcopter. The speed of the propellers was varied to examine how the HERM lines would change. Between 0 s and 14 s, the quadcopter was started and all propellers were simultaneously accelerated and decelerated leading to a HERM lines characteristic like for a one-propeller drone. At 14 s, the drone was manoeuvered to the left and the HERM lines split into two different frequencies. This is characteristic for a multi-propeller drone. Because of the four propeller configuration, two of the propellers start to accelerate and two start to decelerate. At 27 s, the drone was manoeuvered to the right causing the HERM lines to cross and split again. The propellers that were spinning slower before were now spinning faster and vice versa. At 45 s, the drone was switched off.

The multi-frequency detector determined frequencies are shown in Figure 7b. The method was able to measure the chopping and spool frequency at the same time. As expected, only one chopping and spool frequency between 0 s and 14 s was measured. Chopping and spool frequencies are basically determined by the 1st CCP and 2nd CCP, respectively. After 14 s, two non-harmonic chopping and spool frequencies are captured. The two different chopping frequencies are determined by the 1st CCP and 2nd CCP, and the two different spool frequencies are basically determined by the 3rd CCP and 4th CCP. Measuring two non-harmonic chopping or spool rates at the same time indicates that it is a multi-rotor drone. Since the chopping frequency is twice the spool frequency, the drone has to have a two blade propeller.

**Figure 8:**
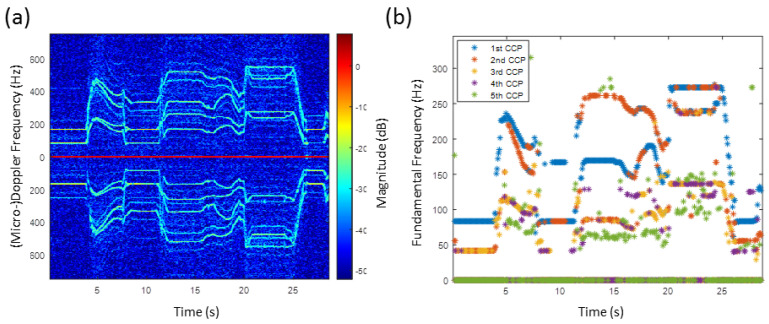
(**a**) HERM lines spectrogram for hexacopter measured by C-band radar; (**b**) corresponding multi-frequency detector result.

Due to the six propeller configuration, the results of the HERM lines spectrogram and of the multi-frequency detector for the hexacopter are very complex for a manoeuvering scenario, see Figure 8. For the two blade propeller drone, the spool frequency is half of the chopping frequency. Moving the drone to the left or to the right direction causes the frequency to be divided into up to three non-harmonic frequencies.

For the first 4.5 s, the drone was hovering stationary leading to straight HERM lines and only one chopping and one spool frequency. In the hovering scenario, all six propellers of the drone were rotating at the same rotation rate. After speeding up and slowing down, the drone was manoeuvered to the left and right recognizable by the crossing of the HERM lines at 7.5 s, 12 s, and 20 s.

The analysis of the multi-frequency detector reveals at least two non-harmonic chopping and spool frequencies between 5 s and 30 s. Unlike for the quadcopter, the chopping and the spool frequency splits into two non-harmonic frequencies when decelerating. Between 5 s and 7.5 s, one propeller chopping frequency is given basically by the 1st CCP and the corresponding spool frequency by the 3rd CCP and a second propeller chopping and spool frequency are given by the 2nd CCP and 5th CCP. Between 12 s and 20 s, one chopping frequency is determined basically by the 2nd CCP and the corresponding spool frequency by the 4th CCP and the second chopping and spool frequency by the 1st CCP and the 3rd CCP. A frequency captured by the 5th CCP can be recognized, but it can not be clearly determined if it is a true chopping or spool frequency. Measuring at least two frequencies indicates that the drone is a multi-propeller drone and measuring at least three frequencies shows that it is most likely as a hexacopter, among the three possibilities. However, note that one has to be careful to interpret three captured chopping or spool frequencies as a hexacopter because a quadcopter drone or drones with more than six propellers can also lead to three different propeller rotation rates under certain conditions.

### 3.4. Multi-Frequency Analysis Using W-Band Radar

**Figure 9:**
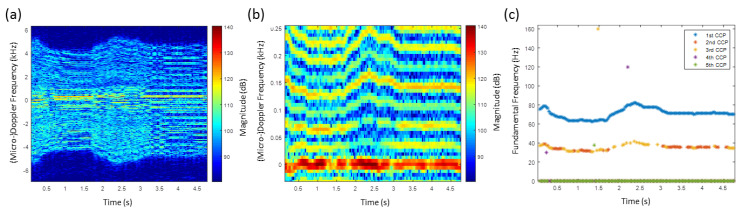
(**a**) HERM lines spectrogram for one-propeller helicopter measured by W-band radar; (**b**) zoomed into the HERM lines spectrogram showing individual HERM lines; (**c**) ccorresponding multi-frequency detector result.

For the one-propeller helicopter, the HERM lines spectrogram and the corresponding fundamental frequency estimate measured by W-band radar are shown in Figure 9. As expected from the one-propeller configuration, Figure 9b shows equally separated HERM lines without any crossings of the lines. Compared to the HERM lines spectrogram in Figure 6, the number of HERM lines is much higher. Because the higher the radar frequency or the smaller the radar wavelength, the higher is the number of HERM lines within the maximum micro-Doppler frequency bandwidth for the same propeller specifications. The Log harmonic summation method reveals only one fundamental frequency at each measuring time. The SNR of this radar is higher so the frequency tracking is better than the C-band radar.

**Figure 10:**
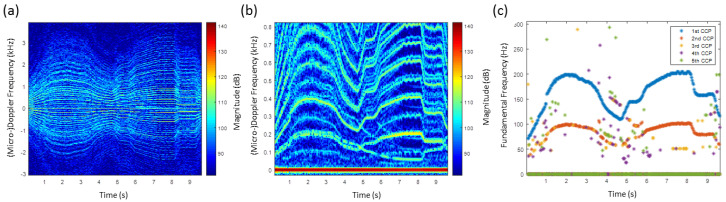
(**a**) HERM lines spectrogram for quadcopter measured by W-band radar; (**b**) zoomed into the HERM lines spectrogram showing individual HERM lines; (**c**) corresponding multi-frequency detector result.

The HERM lines spectrogram for the quadcopter drone are shown in Figure 10. Because of the four-propeller configuration, manoeuvering the quadcopter to the left or to the right side leads to two different propeller rotation rates. The quadcopter was sped up at 4 s and slowed down at 5 s again. As for the results of the C-band radar, the four propellers of the drone do not slow down with the same rotation rate, which can be seen in the splitting of the HERM lines at 5 s, see Figure 10b. At 9 s, the drone was turned to the left.

Unlike the results of the C-band radar in Figure 7, the multi-frequency detector captured clearly just one chopping and one spool frequency, see Figure 10c. A second chopping and spool frequency might be found between 4 s and 5.5 s, but not between 5.5 s and 10 s as expected from the corresponding HERM lines spectrogram. The SNR of the second non-harmonic frequency seems to be too low to be recognized clearly by the multi-frequency detector.

**Figure 11:**
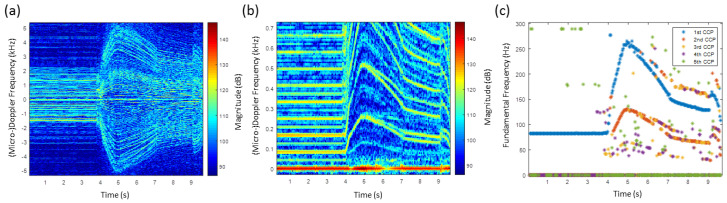
(**a**) HERM lines spectrogram for hexacopter measured by W-band radar; (**b**) zoomed into the HERM lines spectrogram showing individual HERM lines; (**c**) corresponding multi-frequency detector result.

The hexacopter HERM lines spectrogram is shown in Figure 11a,b. For the first 4 s, the propeller rotation rate was constant and similar for all six propellers. Then, the rotation was sped up and slowed down again until 9 s. At 9 s, the drone was turned to the left. After speeding up, at 4.5 s, a dividing and, at 9 s, a crossing of the HERM lines can be clearly recognized. The results of the C-band radar in Figure 8 also revealed a splitting of the HERM lines while slowing down.

Using the multi-frequency detector (see Figure 11c), just one chopping frequency between 0 s and 4 s and above 5 s two different chopping and spool frequencies are determined. The frequency detected basically by the 1st CCP and 2nd CCP are the chopping and spool frequencies, respectively. The captured frequencies between 6 s and 9 s dominated by the 3rd and 4th CCP at about 170 Hz and 90 Hz are probably the chopping and spool frequencies of a second propeller, which gives strong evidence for a multi-propeller drone. The frequencies captured between 4 s and 8 s at about 50 Hz by the 3rd, 4th, and 5th CCP might be false detection.

## 4. Conclusions

Micro-Doppler spectrograms of three different types of drones, namely helicopter, quadcopter, and hexacopter were investigated in an emulated manoeuvering scenarios from data collected using two off-the-shelf radars, the C-band Xethru X4 UWB CW radar, and the W-band TI AWR1642 FMCW radar. The focus was to explore HERM lines (long window STFT) and their micro-Doppler features. HERM lines analysis is more resilient for low SNR and a much lower radar PRF is necessary than for the short window STFT micro-Doppler analysis.

As a new approach, HERM lines were first disentangled as a combination of spool and chopping lines. Spool and chopping lines are proposed as new drone micro-Doppler features for rotary drone detection and classification.

In order to estimate the spool and chopping frequencies of a drone propeller, an analysis of the log harmonic summation algorithm is carried out. This robust method for determining the fundamental frequency from a HERM lines spectrogram was extended to a multi-frequency detector. The multi-frequency analysis method is able to capture the changes of the rotation rate of multiple propellers at the same time.

The multi-frequency detector was applied to the measured spectrograms of drones in laboratory condition manoeuvering scenarios that were emulated in a laboratory setting by varying propeller rotation speeds. It was shown that it is possible to capture multiple chopping and spool frequencies from the same spectrogram and to determine the number of blades per propeller and the multiple propeller rotation rates as a function of time while the drone is manoeuvering. The proposed multi-frequency detector can potentially be used to distinguish a drone from a bird and, if identified as a drone, to determine if it has a single propeller or multiple propellers. These HERM line details provide information that can be utilized in classification and identification of drones. In future work, we will investigate machine learning techniques using HERM line information as features in a classifier.

Finally, note that the HERM line spectrum is also a function of the aspect angles, i.e., depends on the elevation and azimuth angles between the radar and the drone. Investigation of HERM lines of aspect angles will be carried out in future work.

## Figures and Tables

**Table 1:** Specifications for XeThru X4 C-band and TI AWR1642 W-band radars.

	Xethru X4	TI AWR1642
TX center frequency (GHz)	7.29	77
Range Resolution (cm)	5.25	15
Max Range (m)	10	45
Max unambiguous velocity (m/s)	27.83	15.43
RX gain (dB)	12.7	30
PRF (kHz)	1.5	26.3

## Abbreviations

The following abbreviations are used in this manuscript:

HERM	Helicopter Rotor Modulation
JEM	Jem Engine Modulation
LoS	Line of Sight
STFT	Short Time Fourier Transformation
CCP	Cross-Correlation Peak
SNR	Signal-to-Noise Ratio
cw	clockwise
ccw	counterclockwise
PRF	Pulse Repetition Frequency
